# Arrhythmias including atrial fibrillation and congenital heart disease in Kleefstra syndrome: a possible epigenetic link

**DOI:** 10.1093/europace/euae003

**Published:** 2024-01-09

**Authors:** Sunil K Vasireddi, Tanja Zdolsek Draksler, Arianne Bouman, Joost Kummeling, Matthew Wheeler, Chloe Reuter, Siddharth Srivastava, Jacqueline Harris, Paul G Fisher, Sanjiv M Narayan, Paul J Wang, Nitish Badhwar, Tjitske Kleefstra, Marco V Perez

**Affiliations:** Division of Cardiovascular Medicine, Cardiac Arrhythmia Center, Stanford University, 300 Pasteur Drive, Stanford, CA 94305, USA; Cardiovascular Medicine, University of Wisconsin School of Medicine and Public Health, Madison, WI, USA; Centre for Knowledge Transfer in Information Technologies, Jozef Stefan Institute, Ljubljana, Slovenia; IDefine Europe, Ljubljana, Slovenia; Department of Human Genetics, Donders Institute for Brain, Cognition and Behaviour, Radboud University Medical Center, Nijmegen, The Netherlands; Department of Human Genetics, Donders Institute for Brain, Cognition and Behaviour, Radboud University Medical Center, Nijmegen, The Netherlands; Division of Cardiovascular Medicine, Cardiac Arrhythmia Center, Stanford University, 300 Pasteur Drive, Stanford, CA 94305, USA; Stanford Center for Inherited Cardiovascular Diseases, Stanford University, 300 Pasteur Drive, Stanford, CA 94305, USA; Stanford Center for Undiagnosed Diseases, Falk Cardiovascular Research Center, Stanford University, 870 Quarry Road, Palo Alto, CA 94305, USA; Stanford Center for Inherited Cardiovascular Diseases, Stanford University, 300 Pasteur Drive, Stanford, CA 94305, USA; Stanford Center for Undiagnosed Diseases, Falk Cardiovascular Research Center, Stanford University, 870 Quarry Road, Palo Alto, CA 94305, USA; Department of Neurology, Boston Children’s Hospital, Harvard Medical School, Boston, MA, USA; Department of Neurology and Neurogenetics, Kennedy Krieger Institute, Johns Hopkins Medical Institutions, Baltimore, MD, USA; Department of Neurology, Lucile Packard Children’s Hospital, Stanford University, Stanford, CA, USA; Division of Cardiovascular Medicine, Cardiac Arrhythmia Center, Stanford University, 300 Pasteur Drive, Stanford, CA 94305, USA; Division of Cardiovascular Medicine, Cardiac Arrhythmia Center, Stanford University, 300 Pasteur Drive, Stanford, CA 94305, USA; Division of Cardiovascular Medicine, Cardiac Arrhythmia Center, Stanford University, 300 Pasteur Drive, Stanford, CA 94305, USA; Department of Human Genetics, Donders Institute for Brain, Cognition and Behaviour, Radboud University Medical Center, Nijmegen, The Netherlands; Centre of Excellence for Neuropsychiatry, Vincent van Gogh Institute for Psychiatry, Venray, The Netherlands; Division of Cardiovascular Medicine, Cardiac Arrhythmia Center, Stanford University, 300 Pasteur Drive, Stanford, CA 94305, USA; Stanford Center for Inherited Cardiovascular Diseases, Stanford University, 300 Pasteur Drive, Stanford, CA 94305, USA; Stanford Center for Undiagnosed Diseases, Falk Cardiovascular Research Center, Stanford University, 870 Quarry Road, Palo Alto, CA 94305, USA

**Keywords:** Kleefstra syndrome, Epigenetic mutation, Premature atrial arrhythmias, Atrial fibrillation, Congenital/structural heart disease, Pulmonary vein isolation in young

## Abstract

**Aims:**

Kleefstra syndrome (KS), often diagnosed in early childhood, is a rare genetic disorder due to haploinsufficiency of *EHMT1* and is characterized by neuromuscular and intellectual developmental abnormalities. Although congenital heart disease (CHD) is common, the prevalence of arrhythmias and CHD subtypes in KS is unknown.

**Methods and results:**

Inspired by a novel case series of KS patients with atrial tachyarrhythmias in the USA, we evaluate the two largest known KS registries for arrhythmias and CHD: Radboudumc (50 patients) based on health record review at Radboud University Medical Center in the Netherlands and GenIDA (163 patients) based on worldwide surveys of patient families. Three KS patients (aged 17–25 years) presented with atrial tachyarrhythmias without manifest CHD. In the international KS registries, the median [interquartile range (IQR)] age was considerably younger: GenIDA/Radboudumc at 10/13.5 (12/13) years, respectively. Both registries had a 40% prevalence of cardiovascular abnormalities, the majority being CHD, including septal defects, vascular malformations, and valvular disease. Interestingly, 4 (8%) patients in the Radboudumc registry reported arrhythmias without CHD, including one atrial fibrillation (AF), two with supraventricular tachycardias, and one with non-sustained ventricular tachycardia. The GenIDA registry reported one patient with AF and another with chronic ectopic atrial tachycardia (AT). In total, atrial tachyarrhythmias were noted in six young KS patients (6/213 or 3%) with at least four (three AF and one AT) without structural heart disease.

**Conclusion:**

In addition to a high prevalence of CHD, evolving data reveal early-onset atrial tachyarrhythmias in young KS patients, including AF, even in the absence of structural heart disease.

## Graphical Abstract

**Graphical Abstract:**
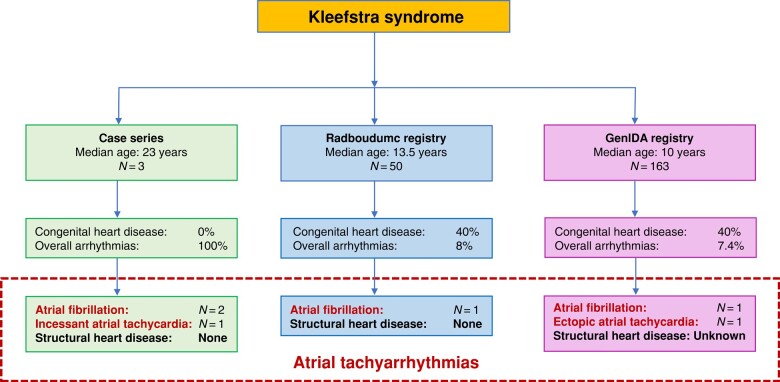

**What’s new?**

- The largest database of patients with Kleefstra syndrome (KS) compiled to date reveals a high prevalence of early-onset atrial arrhythmias and congenital structural heart disease.
- A significant number of KS patients are diagnosed with atrial tachyarrhythmias, including atrial fibrillation (AF), incidentally in their late teen years into their twenties, even in the absence of underlying structural heart disease, suggesting a possible novel epigenetic mechanism for AF.
- Regular electrocardiograms or ambulatory rhythm monitoring in KS patients is advised.

## Background

Kleefstra syndrome (KS), often diagnosed in early childhood, is a rare genetic disorder caused by a sub-telomeric deletion in chromosome 9 (9q34.3) or by a rare variant in euchromatic histone lysine methyltransferase 1 (*EHMT1*), leading to haploinsufficiency of *EHMT1*, and is characterized by the core phenotype of hypotonia, developmental delay/intellectual disability, and distinct facial features.^1,2^ Kleefstra syndrome has also been associated with neuromuscular and skeletal abnormalities, delayed or impaired speech, seizures, as well as behavioural and sleep abnormalities.^1–5^ Congenital heart disease (CHD) is frequently reported in KS patients (10–50%) and primarily includes structural defects such as atrial septal defects (ASD), ventricular septal defects (VSD), aortic coarctation, bicuspid aortic valve, pulmonary valve stenosis, persistent foramen ovale, and patent ductus arteriosus.^1–7^ Conotruncal cardiac defects including tetralogy of Fallot and transposition of great vessels have also been reported.^6–8^ Additionally, individual case reports have shown aberrant muscle bundle in the left ventricle,^1^ hypoplastic left heart,^9^ and pulmonary hypertension^10^ in KS patients.

The *EHMT1* gene plays a vital role in the epigenomic modulation of a myriad of other genes, including those that regulate cardiomyocyte cell cycle and maturation from a neonatal to an adult state.^11,12^ Given that neonatal and mature cardiomyocytes possess significant electrophysiological differences, including calcium regulation and metabolism, it is plausible that the *EHMT1* gene might play a role in arrhythmogenesis indirectly owing to its modulation of cell maturity. Mechanistic investigations assessing the role of epigenetics, particularly post-translational histone modifications and DNA methylation, towards cardiac development and CHD have been increasingly gaining scientific interest.^12–14^ Thus far, in animal and cellular studies, *EHMT1* mutations have been explicitly linked to malformation of the atrioventricular septum,^13^ and modulation of *EHMT1* gene expression (microRNA or medication induced) has been shown to play an essential role in either promoting or suppressing pathologic hypertrophy.^14^ While there have been human cellular studies aimed at neurodevelopmental and cognitive implications due to *EHMT1* mutations studying neural morphology and cholinergic function using patient derived induced pluripotent stem cells (iPSCs),^15,16^ there are currently no studies evaluating potential arrhythmogenic mechanisms in KS.

There is also a paucity of data regarding the risk or type of arrhythmias that might be seen clinically in KS patients. While there have been isolated reports of atrial flutter^2^ and one report of atrial fibrillation (AF) that was incidentally discovered in KS patients,^1,2^ the overall prevalence of atrial arrhythmias in KS is unclear. In patients without structural heart disease, AF would be otherwise unexpected in this age group.

Because of the high incidence of structural heart disease in KS, it is recommended to have at least a baseline electrocardiogram (EKG) after the diagnosis is established^2^ (Kleefstra *et al.*, GeneReviews^®^). However, patients are not routinely evaluated with ambulatory rhythm monitoring or EKG at regular intervals. We aimed to systematically measure the prevalence of CHD and arrhythmias that might inform decisions on surveillance and treatment in this population.

## Methods

We describe a case series of three KS patients with atrial arrhythmias (two patients with AF) in the USA, one each at Stanford Arrhythmia Center, Boston Children’s/Harvard Medical School, and Johns Hopkins Children’s Center in no particular order.

Given the rarity of KS diagnosis worldwide, we sought to systematically analyse the two largest known registries of patients with KS. The Radboudumc registry includes all patients referred to Radboud University Medical Center in the Netherlands who were clinically confirmed to have KS and have consented to the use of their de-identified health records.

The GenIDA international registry (https://genida.unistra.fr/) is a ‘participatory’ online registry in seven languages aimed at compiling the genotype and phenotype information of neurodevelopmental disorders worldwide.^17^ Among over 1500 patients within this registry of rare disorders, all 163 patients with reported KS by January 2022 were included in this study. Patients and families (often referred by medical professionals) self-report their KS diagnosis and answer standardized multiple-choice questions, including cardiovascular abnormalities, arrhythmia status, and cardiomyopathy for the specific listed categories (*Table 1*) using an online questionnaire. Free text options are also available to further clarify diagnoses, and information reported here were also used to derive specific structural heart disease or arrhythmia diagnoses where appropriate.

**Table 1:** Prevalence of cardiac concerns along with common types of CHD in KS within Radboudumc and GenIDA registries

Kleefstra syndrome	Cohort	
	Radboudumc	GenIDA
Total included patients	50	163
Age at last examination in years: median (IQR)	13.5 (13)	10 (12)
Gender (male/female)	M: 38%, F: 62%	M: 48%, F: 52%
Abnormality of the cardiovascular system	20 (40%)	65 (40%)
Arrhythmia	4 (8%)	12 (7.4%)
Cardiomyopathy	None reported	2 (1.2%)
Abnormal ventricular septum morphology	6 (12%)	16 (9.8%)
Atrial septal defect	7 (14%)	21 (12.8%)
Tetralogy of Fallot	2 (4%)	0%
Patent foramen ovale	2 (4%)	3 (1.8%)
Patent ductus arteriosus	N/A	9 (5.5%)
Pulmonary valve stenosis	2 (4%)	14 (8.6%)
Bicuspid aortic valve/aortic valve stenosis	None reported	7 (4.3%)
Mitral or tricuspid valve prolapse/insufficiency	2 (4%)	5 (3.1%)
Abnormality of the greater vasculature	None reported	14 (8.6%)

IQR, interquartile range.

To what extent patients with a confirmed KS diagnosis might not be captured in these two registries and how many patient families decline to enrol in a registry remains unclear. We suspect that most, if not all, the patients diagnosed with this rare disorder join one of these two KS-focused registries that are directly associated with Dr Kleefstra: Radboudumc registry if seen at that institution or GenIDA otherwise. We verified that while up to four patients could potentially overlap between the two registries, none of the patients with arrhythmias overlapped between the registries, and that the patients with arrhythmias reported in this study had not been reported previously. Between the two registries, it is estimated that up to eight KS patients (none with known arrhythmias) may have been previously reported in prior publications that primarily focused on assessing neurodevelopmental outcomes.

Institutional Review Board approval was obtained at Radboudumc, exempted at Stanford, and de-identified patient data were provided by other institutions involved per their respective individual institutional protocols.

## Results

### Case series of atrial fibrillation in Kleefstra syndrome

**Figure 1:**
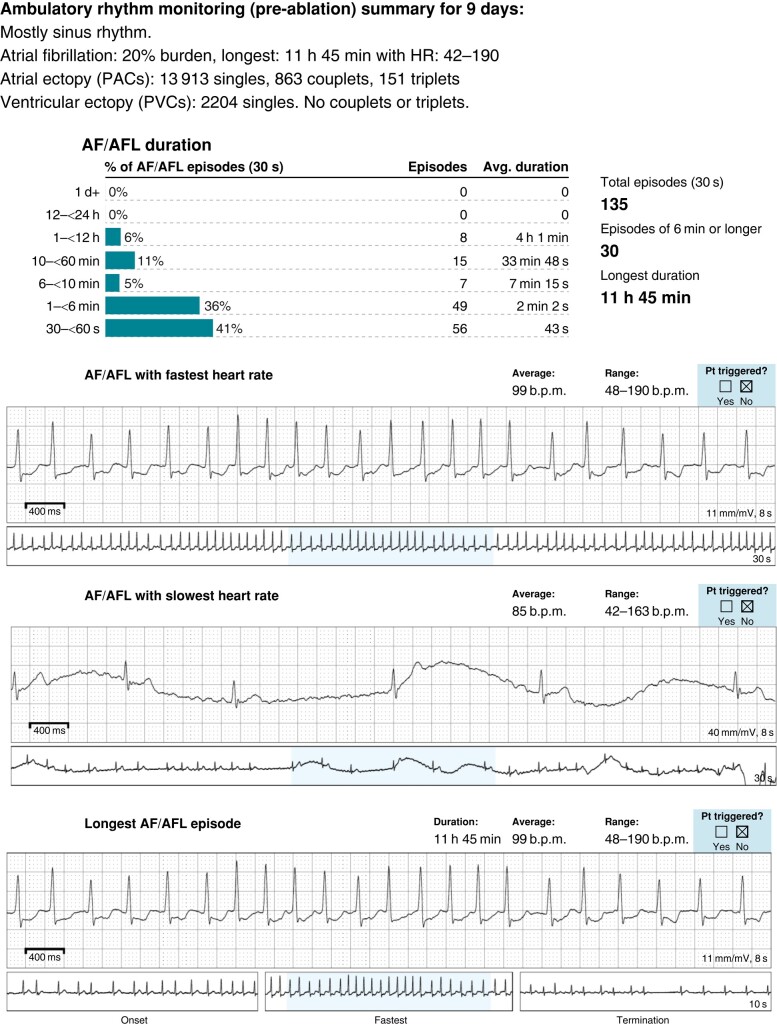
Ambulatory monitoring showing AF in Patient 1 with KS.

The first case (Patient 1) is a 27-year-old man with KS who was first diagnosed with atrial fibrillation at age 23 who presented with fatigue and palpitations with rapid ventricular rates (190 b.p.m.) accompanied by a transient drop in left ventricular function to 45%. His baseline EKG was unremarkable with normal sinus rhythm and occasional premature atrial ectopy (see Supplementary material online, *Figure S1*). He had no structural abnormalities or CHD with an echocardiogram showing normal chamber sizes and function, cardiac magnetic resonance imaging (MRI) showing no scar or late gadolinium enhancement, and cardiac computed tomography (CT) showing normal coronary anatomy with four normal pulmonary veins. He had no reversible causes of arrhythmias identified and given that his symptomatic AF burden rose to above 20% (*Figure 1*) despite treatment with sotalol, he underwent AF ablation. Left atrial mapping revealed a normal left atrial voltage map in sinus rhythm (see Supplementary material online, *Figure S1*) with several triggers in the right inferior pulmonary vein (RIPV) and the left inferior pulmonary vein (LIPV) antrum. Pulmonary vein isolation was performed with additional substrate modification of the posterior wall close to the RIPV antrum. His palpitations had resolved after his AF ablation with a normal EKG, and he did not have any sustained arrhythmia episodes at 8- and 16-month follow-up on 7-day ambulatory monitoring.

Genetic testing in this patient revealed a heterozygous variant of unknown significance (VUS) in the PRKAG2 gene, c.1571T>G (p.Ile524Arg), with a likely pathogenic heterozygous intragenic *EHMT1* duplication of 16 119 bp spanning introns 4–6 (exons 5 and 6). This duplication is predicted to result in a frameshift and premature truncation. Subsequent DNA methylation assay was consistent with the KS episignature, further underscoring the pathogenicity. The patient’s family history is significant for *EHMT1* mosaicism in his asymptomatic father (variant present in blood but not buccal cells).

Two additional young patients with KS with atrial arrhythmias were also identified from across the USA. One patient (Patient 2) was diagnosed with AF incidentally at age 25 during a visit to the dentist, and another patient (Patient 3) at age 17 was admitted for pneumonia and was found to have had incessant atrial tachycardia (AT). Neither of these patients had structural heart disease on cardiac imaging (echocardiography). These patients were medically managed with metoprolol, atenolol, and amiodarone. Inspired by these cases, we systematically evaluated cardiovascular disease, including arrhythmias, in the two largest registries with KS patients.

### Cardiovascular disease in the Radboudumc registry

The Radboudumc expert centre for rare genetic neurodevelopmental disorders in Nijmegen holds a registry with 50 patients with KS who provided consent to use de-identified data. Of these patients, 20 (40%) had cardiovascular abnormalities (*Table 1*), 7 (14%) patients had ASD, 6 (12%) patients had a VSD, and 2 (4%) patients each reported tetralogy of Fallot and pulmonary valve stenosis. None of the patients had left ventricular dysfunction.

*Table 2* shows that in the Radboudumc registry, there were four (8%) patients with arrhythmias, including one patient incidentally diagnosed with paroxysmal AF at the age of 25 years who was treated with metoprolol. There were two reports of supraventricular tachycardias (SVTs): one patient diagnosed with SVT at the age of 18 years (previous normal EKG at the age of 14) who underwent an ablation procedure and was treated with verapamil and another patient diagnosed at age 23 years with an accelerated nodal rhythm who was also treated with verapamil. None of these patients with arrhythmias had CHD on imaging. Additionally, there was one patient with episodes of non-sustained ventricular tachycardia (NSVT) at age 22 years with only a mild valvular insufficiency but a normal left ventricular ejection and no other structural abnormalities.

**Table 2:** Known arrhythmia reports from all the sources including the individual case reports, as well as Radboudumc and GenIDA registries

Source	Cardiac arrhythmia	Treatment	Age at diagnosis (years)	Structural heart disease	Mutation variant	Zygosity (inheritance)
Radboudumc	**Paroxysmal atrial fibrillation**	Metoprolol	25	No	GRCh38 g.137728442C>T *EHMT1* c.736C>T p.(Arg246^a^)	Heterozygous (unknown)
Radboudumc	Supraventricular tachycardia (SVT)	Ablation and verapamil	18	No	GRCh38 g.137798894C>T *EHMT1* c.2587C>T p.(Gln863^a^)	Heterozygous (unknown)
Radboudumc	Paroxysmal SVT (accelerated nodal rhythm)	Verapamil	23	No	GRCh38 g.137776794dup *EHMT1* c.1968dup p.(Gln657Alafs^a^41)	Heterozygous (unknown)
Radboudumc	Non-sustained ventricular tachycardia (NSVT)	Monitoring/expectative	22	Valvular insufficiency normal EF	GRCh38 NC_000009.12 g.pter(203862_534475) del(137507220_138125938)	Heterozygous (unknown)
GenIDA	**Bursts of atrial fibrillation**	Unknown	<29	Not reported	Unknown	Unknown
GenIDA	**Chronic atrial ectopic tachycardia**	Unknown	<19	Not reported	Unknown	Unknown
Patient 1	**Paroxysmal atrial fibrillation, atrial flutter, premature atrial contractions (PACs), NSVT**	Pulmonary vein isolation ablation	23	No	*EHMT1* *16 kb intragenic duplication encompassing exons 5–6* ^a^	Heterozygous (paternal)
Patient 2	**Paroxysmal atrial fibrillation**	Metoprolol	25	No	*EHMT1* IVS22-1G>A c.3259G>A	Heterozygous (*de novo*)
Patient 3	**Unstable SVT** **(incessant atrial tachycardia)**	Atenolol, amiodarone	17	No	*EHMT1* c.3462-10C-G (IVS 24-10C-G)	Heterozygous (unknown)

Atrial arrhythmias are delineated in bold.

^a^Patient also harboured heterozygous PRKAG2, c.1571T>G (p.Ile524Arg) variant of uncertain significance.

### Cardiovascular disease in the GenIDA registry

In the GenIDA registry with 163 KS patients (*Table 1*), there was a prevalence of 40% (65 patients) of cardiovascular abnormalities which is similar to that found in the Radboudumc registry. With respect to CHD, there were 2 (1%) patients with cardiomyopathy, 16 (10%) patients with VSD, 21 (13%) patients with ASD, 9 (5.5%) with patent ductus arteriosus, 26 (16%) patients with valvular disease (bicuspid aortic valve/congenital aortic stenosis, congenital pulmonic valve stenosis, mitral and tricuspid valve insufficiency/prolapse), and 14 (9%) patients with vascular malformations. While most patients with vascular malformations reported aortic dilation, there were isolated reports of one patient with transposition of great vessels, two patients with aortic coarctation, one patient with partial anomalous pulmonary venous return, and four patients with mitral valve prolapse.

In the GenIDA registry, there were 12 (1.4%) patients with self-reported arrhythmias, including 1 patient with AF, 1 patient with chronic ectopic AT, and 1 patient with a ‘T-wave abnormality’ (*Table 2*). Structural heart disease is not reported among these three patients, and it is not clear if it was ever evaluated. The rest of the nine patients self-reported an ‘abnormal EKG’ without further details. Of note, among these nine ‘abnormal EKG’ diagnoses in GenIDA, five patients had combinations of ASD, pulmonary stenosis, cardiovascular malformations, and bicuspid aortic valve (see Supplementary material online, *Table S1*) reported. The true co-existence of clinically meaningful arrhythmias in KS patients with these structural heart disease diagnoses is currently unknown.

*Table 2* shows the compiled data (associated diagnosis, treatment, and KS mutation) from all the patients with arrhythmias studied, including the Radboudumc and GenIDA registries and the three additional case reports from KS patients across the USA. Notably, none of the four patients across the Radboudumc registry and the reported case series with atrial arrhythmias (three AF and one AT) had structural abnormalities on cardiac imaging. Of the remaining two patients with atrial arrhythmias reported in the GenIDA registry (one AF and one AT), although no validated data on imaging are available, none of the patients self-reported structural heart disease.

## Discussion

Evaluation of the prevalence and subtypes of CHD and arrhythmias in KS reported here not only provides novel insights into the diagnosis and treatment of KS patients but also into the role of epigenetics towards possible arrhythmia mechanisms in AF. Kleefstra syndrome has been reported worldwide and identified in each continent in the data reported here (see Supplementary material online, *Table S2*), with the majority of diagnosed patients reported from the USA and Western Europe. The prevalence of KS is likely underestimated due to several challenges. Neurodevelopmental disorders like KS overlap clinically with several others in the autism spectrum and rely on genomic evaluation with chromosomal microarray (CMA) and next-generation sequencing (NGS).^2,18^

However, the clinical spectrum of KS is increasingly better defined as tools like CMA and NGS are more readily available. Arrhythmic disease in KS is also likely underappreciated because patients diagnosed with KS do not routinely undergo regular EKGs or rhythm monitoring. While most patients are likely to have had a baseline EKG in childhood, this is unlikely to adequately capture episodic arrhythmias into adulthood. Not surprisingly, the arrhythmias reported here were incidentally discovered in KS patients between 17 and 29 years of age. It is not entirely clear if this is the age of onset of atrial tachyarrhythmias in KS or if patients in this age group were more likely to undergo an EKG evaluation for neurologic and psychiatric medications.

Kleefstra syndrome patients also have unique barriers to cardiac rhythm screening due to difficulties in patient communication of their complex symptomatology, as well as a low baseline clinical suspicion by most providers currently to associate KS patients’ symptomatology (which might include psychologic and mood deteriorations) with arrhythmias. Hence, KS patients might benefit from routine rhythm screenings with EKG or ambulatory screening to aid in a timely diagnosis and treatment of possible arrhythmias.

Additionally, we also show a high prevalence of several types of congenital structural heart diseases in the largest pool of KS patients to date. These congenital heart defects might predispose KS patients to SVT, ventricular tachycardia (VT), AT, atrial flutter, and AF. Valvular heart disease, cardiomyopathy, and structural anomalies within pulmonary vasculature (with or without lung disease) could promote left-sided AT (focal or macro re-entry) and AF. Hence, detailed cardiac structural imaging assessing anomalous congenital anatomy, including pulmonary vasculature and pulmonary venous return, the presence of cardiomyopathy, scar, abnormal chamber sizes, valvular disease, and history of prior cardiac surgeries, is very important for assessing arrhythmic risk in these patients.

While we do not have such specific imaging information from all the patients’ reported data within the registries, we have shown here a detailed case with physician-confirmed data for KS patient, Patient 1, who had clinically significant AF in his early 20s with a normal heart on imaging (echo, CT, and cardiac MRI) without any known pathology that might explain his AF. Hence, at least in this case and three other young KS patients with AF reported here, the early-onset AF was unrelated to structural heart disease. Overall, new onset AF leading to cardiomyopathy is very rare in this age group, but in the case of Patient 1, AF led to significant clinical deterioration refractory to medical therapy, and the patient benefited from an AF ablation.

This work implies an association between KS and atrial tachyarrhythmias, particularly AF, in young adults. If there is a central arrhythmia mechanism, clinically, we suspect automatic or triggered activity given that one KS patient with AF who underwent ablation was found to have pulmonary vein triggers, and several others are noted to have significant atrial ectopy and SVTs including AT.

It is possible that *EHMT1* either directly (via a channelopathy) or indirectly (via its regulation of cardiac cellular maturation) modulates ion channels and gap junction function and expression. *EHMT1* might also influence autonomics, cardiomyocyte metabolism, energetics, and immune signalling pathways.^19^ By promoting a more neonatal cardiomyocyte state, *EHMT1* mutation might promote arrhythmia mechanisms such as triggered activity and automaticity via calcium or metabolic dysregulation, especially under stress. Alternatively functional or structural re-entry might also be plausible owing to anisotropy, heterogeneous dispersion of repolarization, sub-clinical structural or inflammatory myopathy (that was not seen on MRI), atrial stretch, or early-onset aging, especially at the pulmonary veins and myocyte interfaces (border zone macro re-entry phenomenon). Kleefstra syndrome might also promote arrhythmogenesis by pathologic remodelling of the cardiac extracellular matrix architecture including cardiac mesenchymal cells and their extensive secretome, known to play a role in arrhythmogenesis with or without overt structural disease.^20,21^

## Limitations

There are several limitations when attempting to study such a rare condition with limited available information based on health records at a single institution, patient and family surveys from patients referred from around the world, and select cases at multiple institutions across the USA. Despite best efforts, not all KS patients might be captured and represented in the two registries presented. Furthermore, information from these registries is not comprehensive, is not specifically aimed to target cardiac concerns, and in the GenIDA registry, self-reported conditions were not validated with medical record review.

Despite these limitations, the Radboudumc registry is the largest medically confirmed registry of this very rare disease. The GenIDA registry provides supportive evidence of some of the estimates that are reported, and the small case series presents detailed examples of cases of arrhythmias in young KS patients. This data provide otherwise inaccessible insights into diagnosing, treating, and guiding further focused work on arrhythmias in KS patients.

## Conclusion

We compiled the two largest known KS registries with this rare genetic disorder to reveal a high prevalence of various types of congenital structural heart disease and early-onset arrhythmias. According to a significant number of KS patients diagnosed with atrial tachyarrhythmias incidentally in their young adulthood, EKG and ambulatory monitoring should be incorporated into routine follow-up care. Given that clinically significant AF and AT were noted in six patients (6/213 or 3%) in total with at least four young KS patients (three AF and one AT) without structural heart disease, there is the possibility of a novel epigenetic mechanism for AF which warrants further study, and might help better understand this complex arrhythmia in patients of all ages with or without KS.

## Supplementary Material

euae003_Supplementary_Data

## Data Availability

The data underlying this article are available in the article and in its online Supplementary material. Additional data that support the findings of this study will be shared on reasonable request to the corresponding author.
