# Supplementary figures and images for: Arrhythmias including atrial fibrillation and congenital heart disease in Kleefstra syndrome: a possible epigenetic link
Source: Europace. 2024 Jan 9;26(1):euae003. doi: 10.1093/europace/euae003 (PMC10803030; doi:10.1093/europace/euae003)

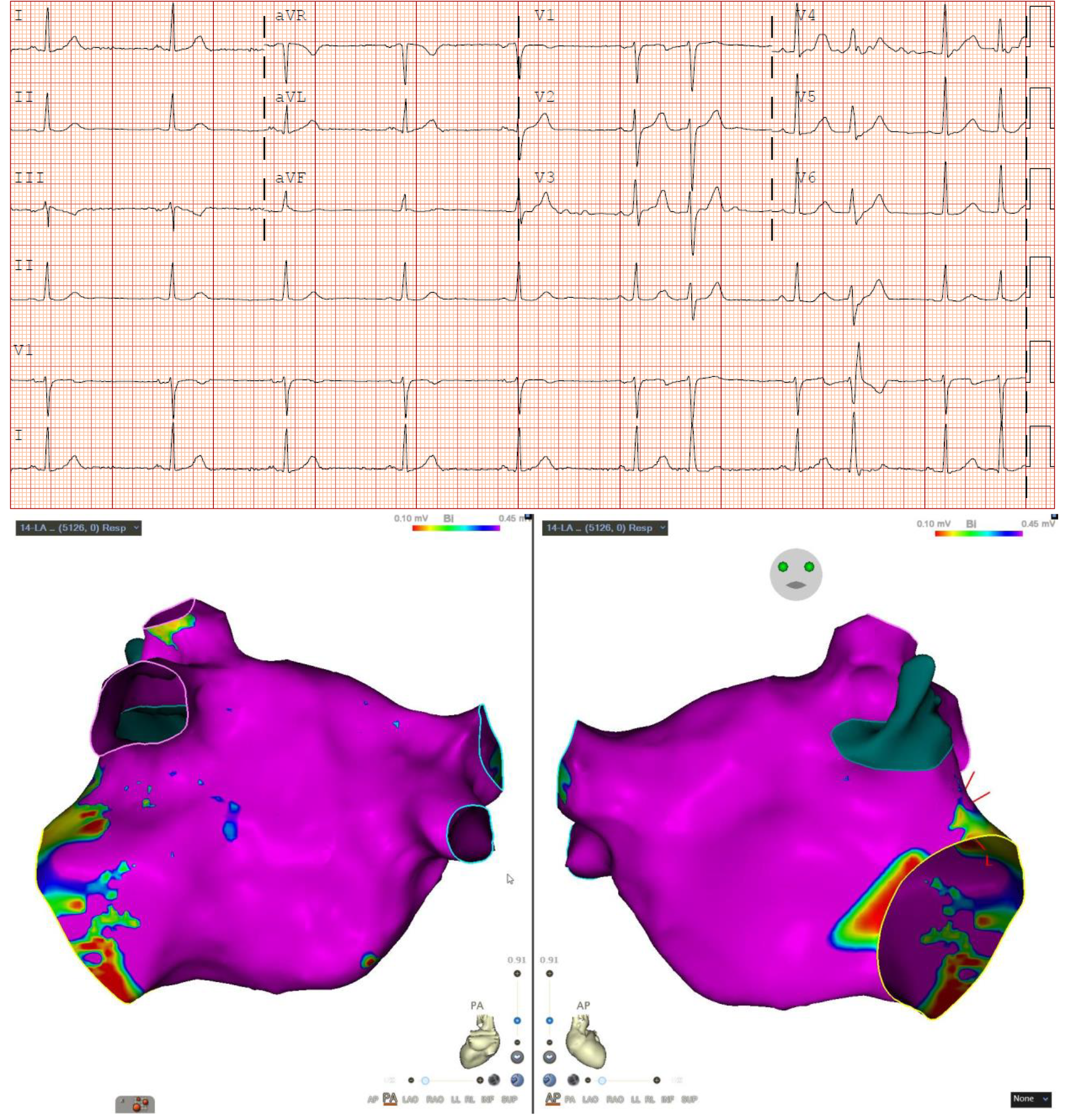

Supplement: euae003_Supplementary_Data [file euae003_supplementary_data.zip › 4-KS_Europace_Figures and Tables-Supplementary Figure1_new.tif]

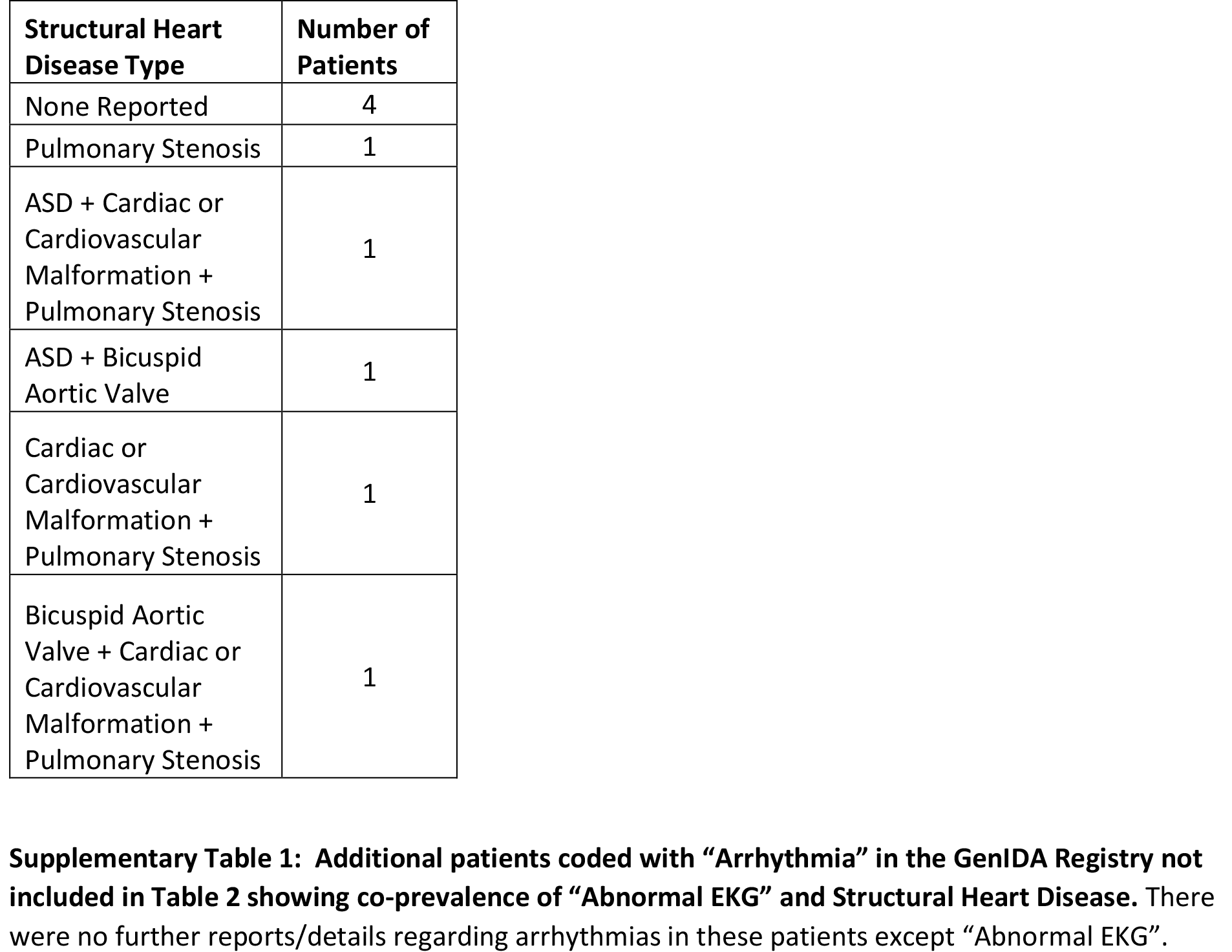

Supplement: euae003_Supplementary_Data [file euae003_supplementary_data.zip › KS_Europace_Figures and Tables-Supplementary Table1.tif]

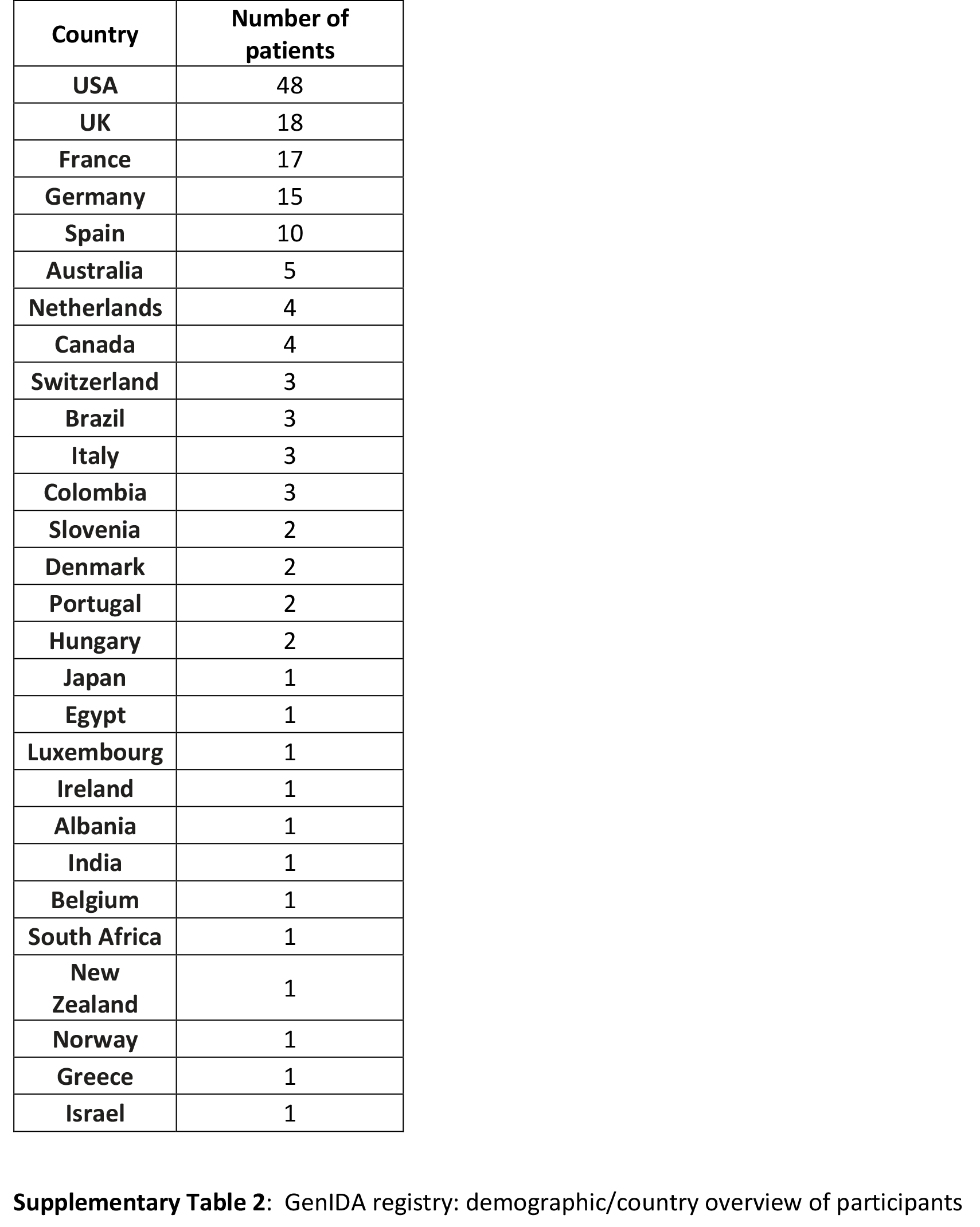

Supplement: euae003_Supplementary_Data [file euae003_supplementary_data.zip › KS_Europace_Figures and Tables-Supplementary Table2.tif]
